# Genetic diversity of *Cryptosporidium* identified in clinical samples from cities in Brazil and Argentina

**DOI:** 10.1590/0074-02760150303

**Published:** 2016-01

**Authors:** Regina Helena Saramago Peralta, Jorge Néstor Velásquez, Flavia de Souza Cunha, María Laura Pantano, Fernando Campos Sodré, Sidnei da Silva, Osvaldo Germán Astudillo, José Mauro Peralta, Silvana Carnevale

**Affiliations:** 1 Universidade Federal Fluminense, Faculdade de Medicina, Departamento de Patologia, Niterói, RJ, Brasil; 2 Hospital Municipal de Infecciosas Dr Francisco Javier Muñiz, Buenos Aires, Argentina; 3 Instituto Nacional de Enfermedades Infecciosas, Administración Nacional de Laboratorios e Institutos de Salud Dr Carlos G Malbrán, Buenos Aires, Argentina; 4 Fundação Oswaldo Cruz, Instituto Nacional de Infectologia Evandro Chagas, Laboratório de Parasitologia, Rio de Janeiro, RJ, Brasil; 5 Universidade Federal do Rio de Janeiro, Instituto de Microbiologia Prof Paulo de Góes, Rio de Janeiro, RJ, Brasil; 6 Consejo Nacional de Investigaciones Científicas y Técnicas, Buenos Aires, Argentina

**Keywords:** Cryptosporidium, genotypes, *gp60* subtypes, PCR

## Abstract

The identification and characterisation of *Cryptosporidium*genotypes and subtypes are fundamental to the study of cryptosporidiosis epidemiology, aiding in prevention and control strategies. The objective was to determine the genetic diversity of*Cryptosporidium* in samples obtained from hospitals of Rio de Janeiro, Brazil, and Buenos Aires, Argentina. Samples were analysed by microscopy and TaqMan polymerase chain reaction (PCR) assays for*Cryptosporidium* detection, genotyped by nested-PCR-restriction fragment length polymorphism (RFLP) analysis of the 18S rRNA gene and subtyped by DNA sequencing of the *gp60* gene. Among the 89 samples from Rio de Janeiro, *Cryptosporidium* spp were detected in 26 by microscopy/TaqMan PCR. In samples from Buenos Aires,*Cryptosporidium* was diagnosed in 15 patients of the 132 studied. The TaqMan PCR and the nested-PCR-RFLP detected *Cryptosporidium parvum*, *Cryptosporidium hominis,* and co-infections of both species. In Brazilian samples, the subtypes IbA10G2 and IIcA5G3 were observed. The subtypes found in Argentinean samples were IbA10G2, IaA10G1R4, IaA11G1R4, and IeA11G3T3, and mixed subtypes of Ia and IIa families were detected in the co-infections. *C. hominis* was the species more frequently detected, and subtype family Ib was reported in both countries. Subtype diversity was higher in Buenos Aires than in Rio de Janeiro and two new subtypes were described for the first time.

*Cryptosporidium* infections in immunocompromised individuals can be asymptomatic or can have severe clinical symptoms, such as profuse diarrhoea, which is usually accompanied by weight loss, malabsorption syndrome, and cholangitis (Putignani & Menichella 2010). In the last two decades, some studies have described a significant risk factor for acquiring intestinal parasitic infections among human immunodeficiency virus (HIV)-infected patients compared with non-HIV controls. Cryptosporidiosis has a high prevalence among intestinal protozoa (Moura et al. 1989, Mohammad et al. 2004, Akinbo et al. 2010).

Laboratory diagnosis of cryptosporidiosis is usually performed by identification of oocysts in stools using an acid-fast stain, but this does not allow species identification, as they are morphologically indistinguishable (Chalmers & Katzer 2013). The identification and characterisation of *Cryptosporidium* species and population variants (genotypes and subtypes) are fundamental to the study of cryptosporidiosis epidemiology and are useful in prevention and control strategies (Putignani & Menichella 2010). Because the oocysts of many species are indistinguishable from one another, molecular methods are essential for identification of the species, genotype, and subtype of *Cryptosporidium* in order to specifically identify the organism responsible for the infection and the source and routes of transmission. The taxonomy of *Cryptosporidium* has been standardised with a guideline that includes morphometric data on oocysts, genetic characterisation, natural, and when feasible, experimental host specificity, and compliance with International Commission on Zoological Nomenclature rules (Ryan et al. 2014).

The methods currently used for detection and species characterisation of*Cryptosporidium* are based on nested-polymerase chain reaction (PCR), PCR-restriction fragment length polymorphism (RFLP), and real-time PCR (Xiao 2010). The genetic markers used are the gene encoding for 18S ribosomal subunit, the *COWP* gene, encoding a protein of the oocyst wall, the *hsp70* gene, which encodes a heat shock protein, internal transcribed spacer (ITS)-1 and ITS-2, the *TRAP* gene, and the gene encoding the glycoproteins GP60 and GP40 (Xiao 2010, Navarro-i-Martinez et al. 2011,Galván et al. 2014). There is no standard genetic *locus* recommended for species identity, but RFLP or sequencing of the 18S rRNA gene provides information about more species than the*COWP* gene *locus* (Muthusamy et al. 2006). Recent work has confirmed the utility of*gp60* sequencing and mini and microsatellite markers in the study of the population structure of *Cryptosporidium* and in understanding the transmission dynamics of infection (Feng et al. 2014). Currently, nearly 20 *Cryptosporidium* species and genotypes have been reported in humans, including *Cryptosporidium hominis*, *Cryptosporidium parvum*, *Cryptosporidium meleagridis*, *Cryptosporidium felis*,*Cryptosporidium canis*, *Cryptosporidium cuniculus*,*Cryptosporidium ubiquitum*, *Cryptosporidium viatorum*, *Cryptosporidium muris*, *Cryptosporidium suis*, *Cryptosporidium fayeri*, *Cryptosporidium andersoni*, *Cryptosporidium bovis*, *Cryptosporidium scrofarum*, *Cryptosporidium tyzzeri*,*Cryptosporidium erinacei* and *Cryptosporidium* I genotypes from horse, skunk, and chipmunk (Xiao 2010, Liu et al. 2014, Ryan & Hijjawi 2015).

In Brazil and Argentina, the prevalence of human cryptosporidiosis varies widely range between 7-24% (Velásquez et al. 1997, Bachur et al. 2008, Barboni et al. 2008, Cardoso et al. 2011, Assis et al. 2013). However, there are few molecular studies of *Cryptosporidium* species and genotypes in humans in these countries where reports about subtypes of*Cryptosporidium* are not described (Carnevale et al. 2010, Meireles 2010,Velásquez et al. 2010, Rolando et al. 2012). Therefore, in the present study, we detected and characterised *Cryptosporidium* species and identified subtypes for the first time in faecal and/or biopsy samples from HIV+ individuals (adults and children) seeking medical attention in public hospitals in the cities of Rio de Janeiro, Brazil, and Buenos Aires, Argentina.

## SUBJECTS, MATERIALS AND METHODS

*Sample collection and DNA extraction in Rio de Janeiro* - Ages of the included patients ranged from 20-75 years for adults and one-16 years for children, with a male/female ratio of 59/30. Eighty-nine faecal samples from HIV-infected patients with diarrhoea (82 adults and 7 children of both sexes) were screened for intestinal parasite infections, with special attention to *Cryptosporidium*spp, using a centrifuge-sedimentation technique, with two centrifugations of 2,000 *g*/2 min (Ritchie 1948). The 89 stool samples were collected from 2009-2013 in three different public hospitals in Rio de Janeiro, originating from Antônio Pedro University Hospital [Faculty of Medicine, Fluminense Federal University (UFF)] (n = 77), Jesus Municipal Hospital (n = 7), and Evandro Chagas Institute of Clinical Research (Oswaldo Cruz Foundation) (n = 5). Only samples with a medical request for the detection of *Cryptosporidium* spp were included in this study.

For *Cryptosporidium* oocyst identification, stool samples were subjected to a modified acid-fast staining technique (Ma & Soave 1983). After parasitological exams, all samples were stored at -20ºC until molecular characterisation. This study was conducted with the approval of the Ethical Review Committee for Research of the Faculty of Medicine, UFF, protocol 020/07.

Total genomic DNA was extracted from 300 mL of stool samples using FastDNA^®^ kit (MP Biomedicals) according to the manufacturer’s protocol. Samples were disrupted in an FP120 cell disruptor (MP Biomedicals) at a speed of 5.5 m/s for 10 s. Potential inhibitors were removed by further purification using QIAquick PCR purification kit (Qiagen), following the manufacturer’s instructions, and DNA extracts were stored at -20ºC until further processing.

*Sample collection and DNA extraction in Buenos Aires* - The study population consisted of 132 adult patients of both sexes (113 male/19 female) diagnosed with HIV who attended the Francisco J Muñiz and José María Penna Hospitals complaining of diarrhoea. Serial stool samples from each patient were processed for parasite analysis by examination of wet mount and slides stained using the modified Ziehl-Neelsen technique for cryptosporidia (Henriksen & Pohlenz 1981), and Weber trichrome (Weber et al. 1992) and Gram-chromothrope (Moura et al. 1996), for microsporidia spores. DNA purification from stool samples was carried out by phenol-chloroform extraction according to Velásquez et al. (2010). Briefly, 1 mL of each sample was treated with 200 mL of ether and centrifuged for 5 min at 15,000 *g*. The pellet was re-suspended in 1 mL of 70% ethanol and stored overnight at -20ºC. Then, the sample was centrifuged for 3 min at 15,000*g*, washed twice with 1 mL of bidistilled water by centrifugation for 3 min at 15,000 *g*, and dried at 37ºC. The pellet was incubated for 45 min at 4ºC in 1 mL of sodium hypochlorite (33 g/L). After this step, the material was centrifuged and washed as previously described. The pellet was then re-suspended in 200 µL of phosphate-buffered saline pH 8 with 20 µL of 5% trypsin and incubated overnight at 37ºC at 200 rpm. All of the following steps were performed as previously described (Velásquez et al. 2010). Biopsy samples from the duodenum were collected by upper gastrointestinal endoscopy, fixed in formaldehyde, paraffin-embedded and stained with Giemsa and haematoxylin-eosin (Carey et al. 2004). Another biopsy specimen was collected in saline solution, stored at -20ºC and employed for DNA extraction and purification according to Velásquez et al. (2010). The research protocol was approved by the Ethical Committee for Research, Hospital Francisco J Muñiz, protocol 274.

The DNA recovery was above 10 pg, measured in a Qubit^®^ fluorometer (Invitrogen, USA).

*TaqMan PCR assays* - The TaqMan PCR procedure combined a duplex reaction for the detection of *Cryp- tosporidium* species and*C. parvum* and a simple reaction for the detection of *C. hominis*, as described previously (Jothikumar et al. 2008). The assays were performed with a 7500 Real-Time PCR System (Life Technologies, USA). Each 20-μL duplex reaction (to identify*Cryptosporidium* species and *C. parvum*) contained 10 μL of 2X Platinum Quantitative PCR SuperMix-UDG (Invitrogen), 100 nM of each probe (JVAP 18S and JVAGP2), 250 nM of each primer (JVAF, JVAR, JVAGF, and JVAGR), and 5 μL of DNA. For the *C. hominis* assay, each 20-μL reaction contained 10 μL of 2X Platinum Quantitative PCR SuperMix-UDG (Invitrogen), 250 nM of each primer (JVAF, JVAR, JVAGF, and JVAGR), 5 mM MgCl_2_, twice the probe concentration used for the duplex assay (200 nM), and 5 μL of DNA. The*Cryptosporidium* PCR cycling conditions consisted of denaturation at 95ºC for 2 min followed by 45 cycles of denaturation at 94ºC for 10 s, annealing at 55ºC for 30 s, and extension at 72ºC for 20 s. All assays included positive controls (*C. hominis* and *C. parvum*) and negative controls (DNA extracted from faecal samples negative for any parasites). To investigate the presence of inhibitory substances, negative samples were contaminated with around 10 fg of *Cryptosporidium* DNA and subsequently submitted to TaqMan PCR.

*Genotyping analysis* - *Cryptosporidium* species were determined by nested PCR amplifying a 214 bp fragment of the small-subunit 18S rRNA gene and RFLP analysis of the secondary PCR products, using the restriction enzymes*Taq*I (Fermentas, Lifescience) and *Vsp*I (Fermentas, Life Science). Primers and amplification conditions used for PCR-RFLP were adopted from previous publications (Coupe et al. 2005). Reaction mixtures were prepared according to Velásquez et al. (2010), employing 10X buffer containing (NH4)_2_SO_4_, and adding 400 ng/µL of bovine serum albumin. Cycle conditions were as follows: one cycle of 94ºC for 5 min, 39 cycles of a denaturation step at 94ºC for 30 s, an annealing step at 60ºC (58ºC for the 2nd round) for 45 s, and an extension step at 72ºC for 90 s, with a final extension for 10 min at 72ºC.

Enzymatic-digested products were fractionated on a 2% agarose gel and visualised by ethidium bromide staining. All diagnoses were confirmed by RFLP analysis of additional, independent PCR products from the same sample.

Samples that contained *C. parvum* and *C. hominis*were further subtyped by DNA sequencing of the *gp60* gene amplified by a nested PCR following the protocol described by Glaberman et al. (2002). Each sample was amplified at least three times by PCR. Primers AL3531 and AL3533 were used in the primary PCR and primers AL3532 and LX0029 in the secondary PCR. *gp60* products were purified according to the manufacturer’s instructions using a NucleoSpin^®^ Extract II kit (MACHEREY-NAGEL GmbH and Co KG, Germany). Sequencing was carried out in both directions by the sequencing services of Institute of Biophysics Carlos Chagas, Macromolecular Metabolism Laboratory, Federal University of Rio de Janeiro (UFRJ), and Medical Biochemistry Institute, SONDA Laboratory, UFRJ, for the Brazilian samples, and by Macrogen Service, for the Argentinean ones. The nucleotide sequences obtained in this study were aligned with reference sequences retrieved from GenBank and *gp60* sequences. The resulting sequences were edited and aligned with the Bioedit Sequence Alignment Editor 7.0.5.3.

## RESULTS

*Samples from Brazil* - Thirty faecal samples were parasitologically positive in the exams and *Cryptosporidium* sp. oocysts were observed in 17 (19.1%) samples. The other parasites detected in these samples were*Strongyloides stercoralis* (2.2%), *Giardia duodenalis*(2.2%), *Entamoeba coli* (1.1%), *Entamoeba histolytica/Entamoeba dispar* complex (2.2%), *Blastocystis*sp. (5.6%), and *Cystoisospora belli* (1.1%). The results of the dual TaqMan PCR procedure for the stool samples from patients are shown in Table I. The 18S rRNA TaqMan assay detected*Cryptosporidium* in 23 (25.8%) samples. Four samples were amplified in the *C. parvum* assay, 14 samples were amplified as*C. hominis*, one sample was a mixed infection with *C. hominis* and *C. parvum*, and four samples were amplified only as a *Cryptosporidium* spp. Three samples previously determined to be *Cryptosporidium*-positive for microscopy were*Cryptosporidium*-negative using the TaqMan assay. For PCR inhibition control, these samples were contaminated with a known concentration of*Cryptosporidium* DNA and retested by PCR. All spiking samples tested had DNA amplification showing no inhibition effect. Species and subtypes of all 26 samples considered *Cryptosporidium-*positive by TaqMan assay and/or microscopy were analysed by nested-PCR-RFLP for 18S rRNA and nested-PCR for*gp60* (Table I). Eight of these samples did not amplify during 18S rRNA PCR or *gp60* PCR and two amplified only in *gp60* PCR. Nine samples were negative in microscopy and four amplified only for *Cryptosporidium* spp in the TaqMan PCR assay. The RFLP confirmed the result from the TaqMan assay, including the result with a mixed infection. The subtype IbA10G2 was observed in 13 *C. hominis* samples and was the only subtype found in this studied population. In four samples presented *C. parvum*, one was sequenced in which the subtype IIcA5G3 was found. The presence of *C. parvum*was observed only in the children group (4/7).

**TABLE I:** Results of microscopic examination, TaqMan polymerase chain reaction (PCR), PCR-restriction fragment length polymorphism (RFLP) 18S rDNA, and by *gp60* PCR/sequencing of faecal samples from hospitals of the city of Rio de Janeiro, Brazil, for*Cryptosporidium* characterisation

Sample	Microscopy	Real-time PCR	18S rDNA/RFLP	*gp60*/sequencing
Pc1	*Cryptosporidium* spp	*C. hominis*	*C. hominis*	IbA10G2
Pc2	*Cryptosporidium* spp	*C. hominis*	*C. hominis*	IbA10G2
Pc6	Negative	*C. hominis*	No amplification	No amplification
Pc7	Negative	*C. hominis*	*C. hominis*	IbA10G2
Pc8	Negative	*Cryptosporidium* spp	No amplification	No amplification
Pc13	Negative	*Cryptosporidium* spp	No amplification	No amplification
Pc16	*Cryptosporidium* spp	*Cryptosporidium* spp	No amplification	No amplification
Pc17	*Cryptosporidium* spp	No amplification	No amplification	No amplification
Pc20	*Cryptosporidium* spp	*Cryptosporidium* spp	*C. hominis*	IbA10G2
Pc27	Negative	*C. hominis*	*C. hominis*	IbA10G2
Pc28	Negative	*C. parvum*	No amplification	No amplification
Pc44	*Cryptosporidium* spp	No amplification	No amplification	No amplification
Pc46	*Cryptosporidium* spp	No amplification	No amplification	No amplification
Pc52	Negative	*C. hominis*	*C. hominis*	IbA10G2
Pc78	*Cryptosporidium* spp	*C. hominis*	*C. hominis*	IbA10G2
Pc79	*Cryptosporidium* spp	*C. hominis*	*C. hominis*	IbA10G2
Pc80	*Cryptosporidium* spp	*C. hominis*	*C. hominis*	*Cryptosporidium*
Pc81	*Cryptosporidium* spp	*C. hominis*	*C. hominis*	IbA10G2
Pc82	*Cryptosporidium* spp	*C. hominis*	*C. hominis*	IbA10G2
Pc83	*Cryptosporidium* spp	*C. hominis*	*C. hominis*	IbA10G2
Pc84	*Cryptosporidium* spp	*C. hominis*	*C. hominis*	IbA10G2
Pc85	*Cryptosporidium* spp	*C. hominis*	*C. hominis*	*Cryptosporidium*
Pc86	Negative	*C. parvum*	No amplification	IbA10G2
Pc87	Negative	*C. parvum*	No amplification	*Cryptosporidium*
Pc88	*Cryptosporidium* spp	*C. parvum/C. hominis*	*C. parvum*/*C. hominis*	*Cryptosporidium*
Pc89	*Cryptosporidium* spp	*C. parvum*	*C. parvum*	IIcA5G3

*Samples from Argentina* - The ages of 132 patients who participated in this study ranged from 20-50 years. Faecal and/or biopsy samples from 31 patients were positive for parasites and 14 for microsporidia. C*ryptosporidium* was diagnosed in 15 patients (11.4%). The other parasites detected were *C. belli* (9.8%), *G. duodenalis*(5.3%), *S. stercoralis* (3%), and *Cyclospora cayetanensis* (0.8%). Those 15 *Cryptosporidium*-positive samples were analysed by real-time PCR assays and the results are shown in Table II. Five samples were amplified in the*C. parvum* assay, nine samples were amplified for *C. hominis*, and one sample was co-infected with *C. hominis* and *C. parvum*. Species of the 15*Cryptosporidium-*positive samples were analysed by nested-PCR-RFLP for 18S rRNA, revealing nine *C. hominis* infections, four *C. parvum* infections, and two co-infections of both species. The results confirmed the species determined by real-time PCR, with the exception of another case of co-infection that had been detected as *C. parvum*only. The subtypes were analysed by amplification and sequencing of the*gp60* gene in eight samples obtained from biopsy. The subtypes for *C. hominis* were IbA10G2 in three samples and IaA10G1R4, IaA11G1R4, and IeA11G3T3 in one sample each. In two samples with co-infections, mixed subtypes of Ia and IIa were detected.

**TABLE II:** Molecular analyses of *Cryptosporidium* spp positive faecal and biopsy samples from the city of Buenos Aires, Argentina

Samples	Real-time PCR	18S rDNA/ RFLP	*gp60*/ sequencing
1	*C. parvum*	*C. parvum*	NP
2	*C. parvum*	*C. parvum*	NP
4	*C. hominis*	*C. hominis*	NP
6	*C. hominis*	*C. hominis*	IbA10G2
21	*C. hominis*	*C. hominis*	IbA10G2
22	*C. hominis*	*C. hominis*	IbA10G2
23	*C. hominis*	*C. hominis*	IaA10G1R4
24	*C. hominis*	*C. hominis*	IeA11G3T3
25	*C. hominis*	*C. hominis*	IaA11G1R4
27	*C. hominis/ C. parvum*	*C. parvum/ C. hominis*	Ia, IIa
28	*C. parvum*	*C. hominis/ C. parvum*	Ia, IIa
39	*C. parvum*	*C. parvum*	NP
46	*C. parvum*	*C. parvum*	NP
47	*C. hominis*	*C. hominis*	NP
50	*C. hominis*	*C. hominis*	NP

NP: not performed; PCR: polymerase chain reaction; RFLP: restriction fragment length polymorphism.

## DISCUSSION

In the present study, the diversity of *Cryptosporidium* parasites from patients living in the cities of Buenos Aires and Rio de Janeiro was examined. Data on specific genotypes involved in human infections in the region are still limited, and to the best of our knowledge, this is the first report on the study of the *Cryptosporidium* subtypes infecting humans performed in Argentina and Brazil.

The results showed that *C. hominis* was detected more frequently than*C. parvum* in HIV-infected patients from both areas, but co-infections of both species were also present. *C. parvum* and*C. hominis* have been reported as the most common species causing infections in HIV-infected people in developed countries [France, Switzerland, Spain, and the United States of America (USA)] and developing countries (Jamaica, Kenya, Malaysia, Peru, Portugal, South Africa, South India, Thailand, and Vietnam) (Gatei et al. 2003, Zaidah et al. 2008). The results of the present work appear to be consistent with those in Peru, Australia, Kenya, South Africa, Thailand, the USA, and Vietnam, where *C. hominis* is reported to be the more common causative agent of cryptosporidiosis in immunocompromised people (Cama et al. 2007, Widmer & Sullivan 2012). *Cryptosporidium*subtyping at the *gp60* level in HIV infected patients has been carried out in a few countries (Xiao & Feng 2008).

Different types of molecular techniques have been used in the differentiation of*Cryptosporidium* species/genotypes, with the *SSU*rRNA-based tools being the most employed, especially PCR-RFLP (Xiao 2010). The use of this gene is mainly due to the presence of semi-conserved and hyper-variable regions in a multi-copy. In our study, we applied this technique for species identification, and it was also useful for co-infection detection. All positive samples by microscopy were analysed by a previously described TaqMan PCR assay that allowed differentiation of *C. hominis* and *C. parvum* at the species level. According to our results, both methods could be employed simultaneously to improve and confirm the results. Molecular methods for detecting *Cryptosporidium* in clinical specimens have been shown to be more sensitive than conventional microscopy (Chalmers & Katzer 2013, Yang et al. 2013). The TaqMan procedure had a specificity of 94% for detecting *Cryptosporidium* in clinical specimens and a sensitivity that is better than conventional microscopy and comparable to other molecular methods used for confirmatory identification of*Cryptosporidium* species (Jothikumar et al. 2008).

Of four samples that amplified only as a *Cryptosporidium* spp in TaqMan PCR, one also amplified in nested-PCR *gp60* and the subtype was defined as IbA10G2. The others three samples were probably different species of*Cryptosporidium* and additional analysis is necessary for confirmation. We did not have success in amplifying the 18S and *gp60*gene in these samples.

Given that *C. hominis* and *C. parvum* were responsible for the *Cryptosporidium* infections in the HIV-infected patients studied here, a further evaluation of the genetic variation within these two species was carried out by DNA sequence analysis of the 60-kDa glycoprotein gene. This single-copy gene has tandem repeats of the serine-coding trinucleotide TCA, TCG, or TCT at the 5’ end of the gene, which vary in number, and there are extensive sequence differences in the nonrepeat regions, which categorise each species to several subtype families (Strong et al. 2000, Xiao 2010).

Until now, *C. hominis* had nine *gp60* subtype families, Ia-Ij (Nichols et al. 2014). The subtype family Ib is dominant and broadly distributed, with more than 70% of records referencing this subtype (Jex & Gasser 2010), followed by Ia, Id, and Ie, with less than 10% each. Additionally, there is variation in subtype richness, diversity, and distribution within each of the *gp60* subtype families of *C. hominis*. The subtype family Ib is represented by nine subtypes, dominated by IbA10G2, which is distributed on all inhabited continents. This subtype has been implicated in some waterborne and foodborne outbreaks of human cryptosporidiosis (Sulaiman et al. 2001, Glaberman et al. 2002, Cohen et al. 2006,Widerström et al. 2014). Subtype IbA10G2 has been found in approximately 50% of *C. hominis*-associated outbreaks in the USA (Xiao & Ryan 2004). In a longitudinal study in Lima, Peru, IbA10G2 was more virulent than other*C. hominis* subtypes (Cama et al. 2008).

In samples from Rio de Janeiro, only one subtype of *C. hominis* was identified, corresponding to IbA10G2. Instead, in samples from Buenos Aires, three*C. hominis* subtype families were detected (Ib, Ia, and Ie). The IbA10G2 was the predominant subtype, but three others were identified, namely IaA10G1R4, IaA11G1, and IeA11G3T3. Although the sample size is small, homogeneity within the *C. hominis* at the *gp60locus* is unusual, as subtype families such as Ia and Id are usually equally abundant in most developing countries (Leav et al. 2002, Peng et al. 2003, Cama et al. 2008, Valenzuela et al. 2014). It has been shown that there are differences in the molecular epidemiology of *C. hominis* between developing and developed countries. In nonindustrialised countries, a greater variety of *C. hominis*subtypes have been reported with multiple subtype families and multiple subtypes within families Ia and Id. Much less genetic diversity is observed in*C. hominis* in some industrialised countries, where most*C. hominis* infections are caused by the Ib subtype family, with the majority of these cases having the IbA10G2 subtype (Xiao 2010). Recently, the subtype IbA10G2 was also prevalent in 161 cryptosporidiosis cases in two hospitals in Barcelona, Spain, corresponding to 90% of all *C. hominis* isolates (Segura et al. 2015). The presence of Ib and IIc subtype families in Brazil may suggest anthroponotic transmission, while the findings from Argentina suggest that zoonotic transmission may play a role.

Among the Ia subfamily, IaA12G1R1 is the most common subtype and has been reported in humans from Japan, Nepal, Pakistan, and Peru (Wu et al. 2003, Cama et al. 2008, Chalmers 2008). Among this subfamily, two subtypes were identified in the Argentinean samples, belonging to IaA10G1R4 (GenBank KT381976) and IaA11G1R4 (GenBank KT381977), and both have not been previously described.

Subtype family Ie has low subtype richness and diversity, as most Ie human infections in developing countries are caused by IeA11G3T3 (Sharma et al. 2013, Adamu et al. 2014, Valenzuela et al. 2014). Our study in Buenos Aires identified this subtype in one sample.

Regarding *C. parvum*, *gp60* subgenotyping for Brazilian patients identified only the IIcA5G3 lineage in one sample. The subtype family IIa is globally distributed and the most common *C. parvum*infection in humans (Xiao & Feng 2008). The subtype families IIc and IId are also common and broadly distributed. The other known families, IIb and IIe-k, are rare (Jex & Gasser 2010). Eibach et al. (2015)described that all identified *C. parvum* and *C. hominis* subtypes found in Brazilian human samples have not yet been identified in any animal samples, suggesting a dominant anthroponotic transmission in the region of Rio de Janeiro. These authors also found similar results in a rural region of Ghana. This type of transmission is expected in the urban region of Rio de Janeiro.

Genotyping by TaqMan PCR and 18S PCR-RFLP, and subtyping by*gp60*-based sequencing revealed mixed infections of*Cryptosporidium* species/subtypes in three samples. In one brazilian sample, *C. hominis* and *C. parvum* were detected for both genotyping assays, but the *gp60* subtyping was not possible to analyse. For the Argentinean samples, co-infection of *C. hominis* and *C. parvum* were detected in two cases, with the subtype families Ia and IIa. There are few records showing mixed infections at the subtyping level (Ajjampur et al. 2010,Sharma et al. 2013), and this is the first report for human samples from Argentina. The interference of the subtypes in the severity of the infection also beginning to be studied and the IbA10G2 subtype has been described as one of the most virulent among the subtypes of *C. hominis* (Li et al. 2013). Subtype family IbA10G2 was reported in both countries, but great subtype diversity was detected in Argentina. So far, only two studies have reported subtyping of*Cryptosporidium* in South America, involving Peruvian children (Cama et al. 2008) and Peruvian HIV-infected persons (Cama et al. 2007). Differentiating the species and subtypes requires the use of high resolution molecular tools, and subtyping is important for understanding population structure. Further information from larger samples is necessary to provide insights on subtype lineages to elucidate the value of geographical/pathogenic variation in*Cryptosporidium* species/genotypes.
